# Universal Access to HIV Treatment versus Universal ‘Test and Treat’: Transmission, Drug Resistance & Treatment Costs

**DOI:** 10.1371/journal.pone.0041212

**Published:** 2012-09-05

**Authors:** Bradley G. Wagner, Sally Blower

**Affiliations:** David Geffen School of Medicine, University of California Los Angeles, Los Angeles, California, United States of America; University of Ottawa, Canada

## Abstract

In South Africa (SA) universal access to treatment for HIV-infected individuals in need has yet to be achieved. Currently ∼1 million receive treatment, but an additional 1.6 million are in need. It is being debated whether to use a universal ‘test and treat’ (T&T) strategy to try to eliminate HIV in SA; treatment reduces infectivity and hence transmission. Under a T&T strategy all HIV-infected individuals would receive treatment whether in need or not. This would require treating 5 million individuals almost immediately and providing treatment for several decades. We use a validated mathematical model to predict impact and costs of: (i) a universal T&T strategy and (ii) achieving universal access to treatment. Using modeling the WHO has predicted a universal T&T strategy in SA would eliminate HIV within a decade, and (after 40 years) cost ∼$10 billion less than achieving universal access. In contrast, we predict a universal T&T strategy in SA could eliminate HIV, but take 40 years and cost ∼$12 billion more than achieving universal access. We determine the difference in predictions is because the WHO has under-estimated survival time on treatment and ignored the risk of resistance. We predict, after 20 years, ∼2 million individuals would need second-line regimens if a universal T&T strategy is implemented versus ∼1.5 million if universal access is achieved. Costs need to be realistically estimated and multiple evaluation criteria used to compare ‘treatment as prevention’ with other prevention strategies. Before implementing a universal T&T strategy, which may not be sustainable, we recommend striving to achieve universal access to treatment as quickly as possible. We predict achieving universal access to treatment would be a very effective ‘treatment as prevention’ approach and bring the HIV epidemic in SA close to elimination, preventing ∼4 million infections after 20 years and ∼11 million after 40 years.

## Introduction

Treating HIV-infected individuals has both a therapeutic and a preventive effect, because treatment reduces viral load. Reducing viral load increases survival, but also decreases the infectivity of the individual. Consequently by treating HIV-infected individuals, HIV infections are prevented and transmission decreases. It is being debated whether to use a universal ‘test and treat’ (T&T) approach as a prevention strategy to control HIV epidemics [1]–[9]. A universal T&T strategy is based on treating all HIV-infected individuals whether they need treatment or not. In resource-constrained countries individuals are not considered to need treatment until their CD4 count has fallen to 350 cells/µL, this generally occurs ∼5–7 years after infection. Unfortunately, universal access to treatment for those in need has yet to be achieved in many countries. Granich and colleagues at the World Health Organization (WHO) have claimed, based on mathematical modeling, that a universal T&T strategy would lead (within a decade) to HIV elimination in South Africa and cost less (over 40 years) than achieving universal access to treatment in that country [5], [10]. Here we refer to the HIV transmission model, used by Granich and colleagues, as the WHO model. We use a modified version of this model, which incorporates greater realism, to predict the impact on the HIV epidemic in South Africa of (i) a universal T&T strategy and (ii) achieving universal access to treatment. We predict the impact on transmission and drug resistance, we also estimate treatment costs. The universal T&T strategy is based on annual HIV testing for the entire population of South Africa (∼30 million adults aged between 15 and 49 years) and providing immediate treatment for all HIV-infected adults regardless of their CD4 cell count (i.e., their need for treatment). We compare our predictions with the WHO's predictions [5], [10].

We began by predicting the impact of treatment on reducing transmission; we quantified the impact (as did the WHO [5], [10]) in terms of the Control Reproduction Number (R_C_). R_C_ is defined as the average number of new infections one infected individual generates during their lifetime, assuming the entire population is susceptible and biomedical and/or behavioral interventions are in place. If interventions can reduce the value of R_C_ to below one it can be concluded that (theoretically) it is possible to eliminate the disease. We calculated the effect of treatment on reducing the value of the R_C_ under a range of assumptions for: (i) the CD4 cell count level at which treatment is initiated, (ii) the frequency at which the population is tested for HIV infection, and (iii) the degree to which treatment reduces infectivity. We used these results to determine whether universal T&T and/or achieving universal access to treatment could (theoretically) lead to HIV elimination in South Africa. As well as analyzing R_C_ we also numerically simulated our transmission model (as did the WHO [5], [10]). We used our simulations to determine whether elimination, if it was possible, could occur within 40 years. We used the WHO definition of elimination: less than 1 new HIV infection occurring per thousand individuals per year [5], [11].

Our transmission model more realistically represents the natural history of HIV infection than the WHO model [5], [10]. Our model includes three stages: primary infection, chronic infection and AIDS. We model viral loads (hence infectivity) to be highest in primary infection, lower in chronic infection and to increase again in AIDS. We assume HIV-infected individuals spend ∼2 months in primary infection, ∼7.5 years in the chronically infected stage and ∼3.5 years in the AIDS stage. The WHO model the natural infection of HIV as four stages [5], [10]. They assume HIV-infected individuals have the same viral load (hence infectivity), and also spend the same amount of time (∼2.75 years), in each of the four stages. Our transmission model also more realistically represents the effect of treatment than the WHO model. We assume, as in the “real-world”, some HIV-infected individuals develop drug resistance on treatment [12], [13]; consequently we model the evolution of acquired resistance and the dynamics of transmitted resistance. Therefore our model can be used to predict the number of individuals who would need second-line regimens. However the WHO transmission model does not include acquired or transmitted resistance [5], [10]. Therefore their model cannot be used to predict the number of individuals who would need second-line regimens. In addition, our modeling differs from the WHO's modeling in terms of the assumption we make with regard to survival time on treatment; see Methods for details. We also investigate the effect of heterogeneity in response to treatment in terms of viral suppression, hence heterogeneity in treatment-induced reduction in infectivity.

## Methods

Our model, like the WHO model [5], [10], specifies the transmission dynamics of an HIV epidemic driven by heterosexual transmission. A flow diagram of the WHO model is shown in Figure S1 in the SM; the WHO model is described in detail in reference 5. For a detailed description of the structure of our model and equations see Section 1 in the Material S1 (SM). In contrast to the WHO model, our transmission model includes: (i) a realistic representation of the natural history of HIV infection, (ii) the evolution of acquired resistance and the dynamics of transmitted resistance, and (iii) a longer (more realistic) survival time for individuals who initiate treatment at the current treatment threshold of 350 cells/µL.

Following the WHO, we made two assumptions regarding the effect of treatment on increasing survival time of HIV-infected individuals. Our first assumption is the same as the WHO's. Specifically, we assume the survival time of an individual who initiates treatment immediately after infection is ∼6 years longer than the survival time of an individual who initiates treatment when their CD4 count has fallen to 350 cells/µL (i.e., at the current treatment threshold) [5], [10]. However our second assumption is very different than the WHO's assumption. They assume (as did Dodd *et al.* [14]) the survival time of a treated individual with a CD4 count of 350 cells/µL is only ∼6 years longer than the survival time of an untreated individual with a CD4 count of 350 cells/µL [5], [10]. Clinical data show this survival time is unrealistically short; HIV-infected individuals on treatment with a CD4 count of 350 cells/µL can survive for several decades [15]. Therefore, for our modeling we assume a longer survival time than the WHO and Dodd *et al.* ([5], [10], [14]). Specifically, we assume treated individuals (in comparison with untreated individuals) have a 62% chance of surviving an additional 20 years or more after their CD4 cell count has fallen to 350 cells/µL [15].

We used our transmission model to derive a mathematical expression for the Control Reproduction Number (R_C_); see Section 2 in the SM. We calculated the value of R_C_ for a range of population-level testing frequencies (6 months to 4 years) and for a range of treatment initiation thresholds defined in terms of the CD4 cell count (100 cells/µL to 800 cells/µL). We also varied the average treatment-induced reduction in infectivity in a population. We used a maximum value of 96% based on the results of the HPTN 052 clinical trial [16]; this trial has shown treating the HIV-infected partner in a discordant couple reduces the probability of transmission by 96%. HPTN 052 was conducted over 20 months and adherence to the treatment regimen was very high. However, not all individuals in a population are likely to adhere to treatment to the same degree as the trial participants. Consequently, we conducted additional analyses assuming there would be heterogeneity in response to treatment (i,e, in the reduction in viral load and hence in infectivity) due to differences in adherence or other factors. To model this heterogeneity in treatment response we assumed the average treatment-induced reduction in infectivity could be 90% or 85%. Using the values of R_C_ we calculated the threshold at which R_C_ equals one and hence determined whether universal T&T and/or achieving universal access to treatment could (theoretically) lead to HIV elimination in South Africa.

We used demographic and epidemiologic data from South Africa to parameterize our model; all model parameter values are given in Table S1 in the SM. We then validated our transmission model by comparing model-generated HIV prevalence to historical HIV prevalence data for South Africa from 1990 to 2010. To generate retrospective prevalence, we simulated our model from the beginning of the HIV epidemic accounting for the effect of heterogeneity in sexual behavior on the initial growth rate of the epidemic.

We used our validated transmission model to predict the impact on the HIV epidemic in South Africa of (i) a universal T&T strategy (based on annual testing) and (ii) achieving universal access to treatment. We did not model any other prevention strategies in addition to treatment to avoid potential confounding effects. Following the WHO, we ran simulations for 40 years. We predicted the: (i) reduction in incidence rates, (ii) cumulative number of infections prevented, (iii) number of individuals in need of first-line regimens, (iv) number of individuals in need of second-line regimens and (v) annual and cumulative treatment costs for both first-line and second-line regimens. We assumed acquired resistance would emerge at a rate of 3% per year in the treated population. We note this is a very low rate, but since this led to very high levels of drug resistance over a 40 year time period we did not examine the impact of higher rates. We assumed resistant strains would be 50% less fit than wild-type HIV strains in terms of transmissibility. We conducted two numerical analyses. We first simulated our model without including the development and transmission of resistance. We then simulated our model including the development of acquired resistance and the dynamics of transmitted resistance. In both numerical analyses we investigated the effect of heterogeneity in viral suppression due to treatment; hence, heterogeneity in treatment-induced reduction in infectivity. We express the heterogeneity by assuming the average heterogeneity in treatment-induced reduction in infectivity is 90% or 85%.

We calculated treatment costs in United States (US) dollars. Following the WHO [10], we used annual per person treatment costs of $751 per year for first-line regimens, annual per person treatment costs of $1,167 for second-line regimens, and a discount rate of 3.5% per year. Because we include the development of resistance in treated individuals in our transmission model we are able to predict the number of individuals in need of second-line regimens over time. Consequently we are able to directly determine the costs for second-line regimens. By modeling resistance, the prevalence of resistance in the treated population in our simulations increases over time. However, the WHO does not include the development of resistance in treated individuals in their transmission model. Hence their model cannot be used to predict the number of individuals in need of second-line regimens and cannot be used to directly determine the costs for second-line regimens. Therefore, to estimate the costs for second-line regimens the WHO made an assumption. They assumed the prevalence of resistance in the treated population would remain at 3% for 40 years.

## Results

Figure S2 in the SM shows the results of model validation; the Figure compares the HIV prevalence generated by our transmission model (blue curve) and historical HIV prevalence data for South Africa (red data points). As can be seen, our model captures the initial timing, growth, and maturing of the HIV epidemic from 1990 to 2010. HIV incidence generated by our transmission model is shown in Figure S3 in the SM.

**Figure 1:**
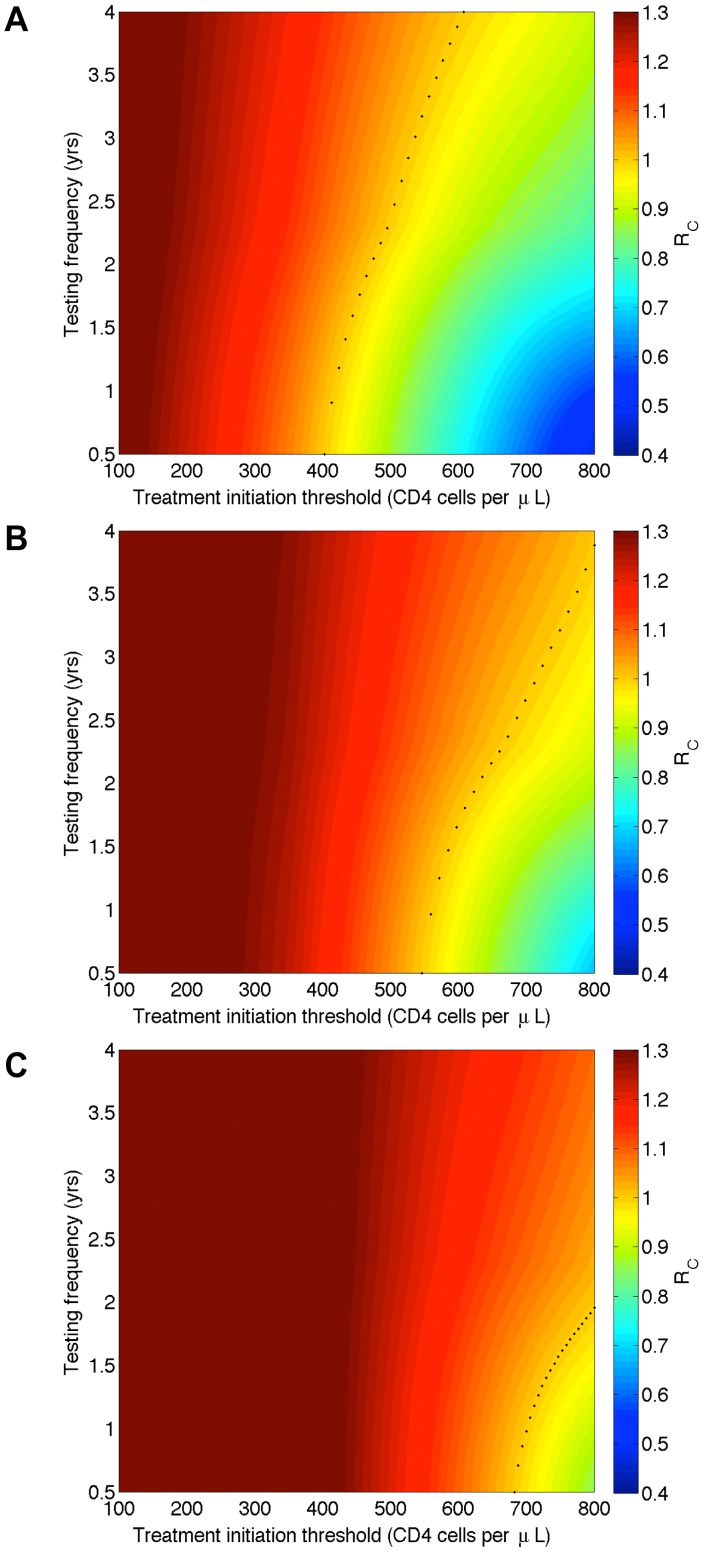
Dependence of the Control Reproduction Number (R_c_) on the average population-level testing frequency for HIV (in terms of years between tests) and the treatment initiation threshold in terms of the CD4 cells/µL. The dotted black line represents the threshold R_c_ = 1; below this threshold (i.e., R_c_<1) elimination is (theoretically) possible. Panels represent the average treatment-induced reduction in infectivity at the population level: (**A**) 96% (**B**) 90% (**C**) 85%.

Results of the R_C_ analysis are shown in the color-coded plots in Figure 1. Colors indicate the magnitude of the R_C_ at that particular pair of parameter values; dark blue is the lowest and dark red is the highest. In each plot the Y-axis shows the frequency (in years) of population-level HIV testing and the X-axis shows the treatment initiation threshold in terms of the CD4 cell count in cells/µL. The dotted black curve in each plot delimits the threshold at which R_c_ equals one; below the curve elimination is (theoretically) possible, and above the curve elimination is not possible. Results in Figure 1 are based on the assumption the average treatment-induced reduction in infectivity is 96% (Figure 1A), 90% (Figure 1B) or 85% (Figure 1C). The treatment initiation threshold necessary to achieve elimination decreases as testing frequency increases (Figure 1A, 1B and 1C). Notably, higher treatment initiation thresholds and more frequent testing are required to achieve elimination when the effect of treatment on reducing infectivity is less than the 96% observed in the HPTN 052 trial, compare Figure 1B and 1C with 1A.

If the treatment initiation threshold is high (CD4 count ∼600 cells/µL) and the reduction in infectivity is very high (96%), elimination could occur as long as the population is tested for HIV at least once every 4 years (Figure 1A). At this high treatment initiation threshold, even if the reduction in infectivity is lower (90% (Figure 1B) or 85% (Figure 1C)) elimination could occur if population-level HIV testing occurs more than once every 2 years. The results shown in Figures 1A, 1B and 1C imply that implementing a universal T&T strategy could (theoretically) lead to HIV elimination in South Africa as long as the treatment-induced reduction in infectivity is greater than 85% and population-level testing is fairly frequent. Notably, the results in Figure 1A also show that achieving universal access to treatment (based on the current treatment initiation threshold) coupled with annual testing could bring the South African HIV epidemic close to elimination. For this to occur, the average treatment-induced reduction in infectivity would have to be high, 96% (Figure 1A).

**Figure 2:**
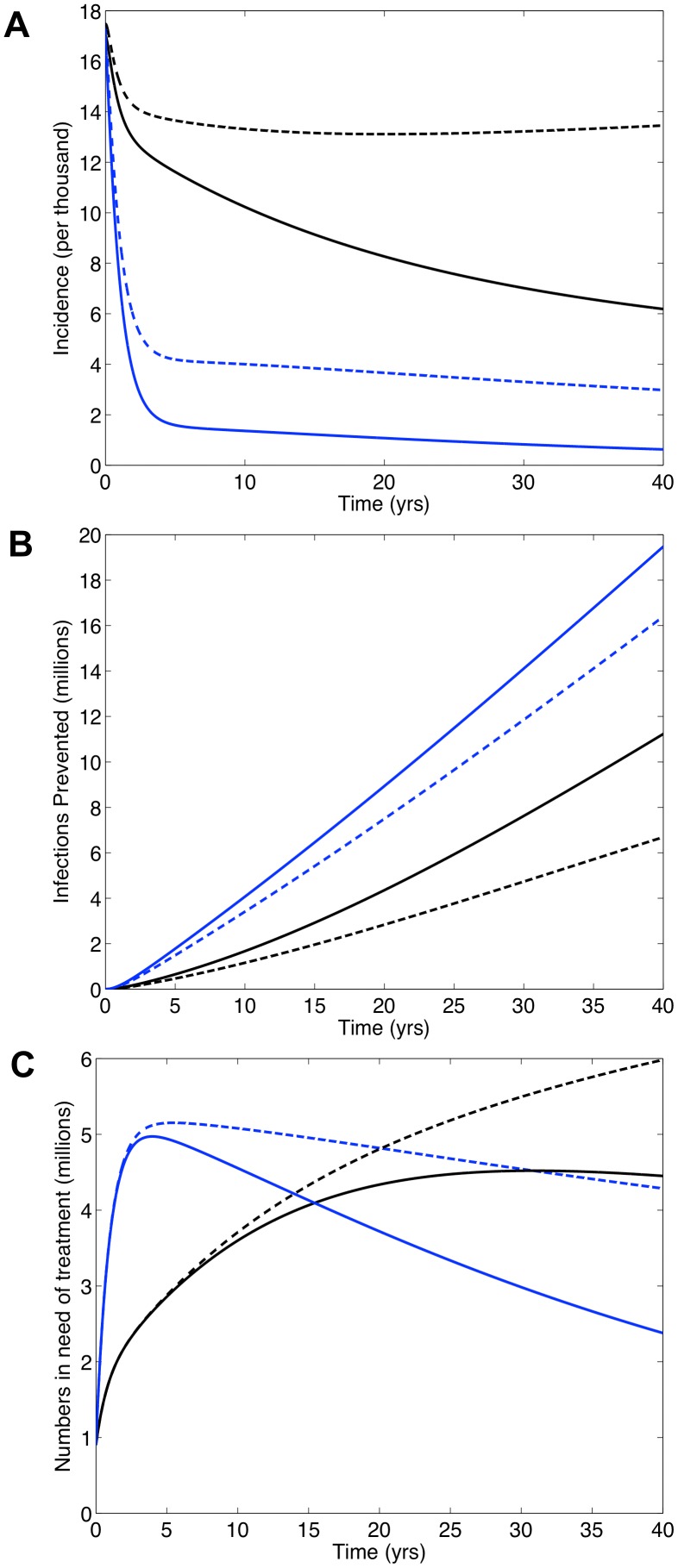
Predictions for South Africa generated from our transmission model if universal access to treatment is achieved (black curves) or a universal T&T (with annual testing) strategy (blue curves) is implemented. The dynamics of acquired and transmitted drug resistance are not included in these simulations. Solid lines denote the case where the treatment-induced reduction in infectivity is 96% and dashed lines denote the case where the reduction is 85%. Panels show (**A**) annual incidence over time, (**B**) number of infections prevented over time and (**C**) number of individuals in need of treatment over time.

Figure 2 shows the predictions for South Africa generated by our transmission model: universal access to treatment (black curves), universal T&T with annual testing (blue curves). Solid curves denote an average treatment-induced reduction in infectivity of 96%; dashed curves denote a reduction of 85%. These simulations do not include the development and transmission of drug-resistant strains of HIV; consequently, they can be directly compared with the WHO's simulations which do not include resistance [5], [10]. The universal T&T strategy leads to a dramatic drop in HIV incidence, whether infectivity is reduced by 96% or 85% (Figure 2A). Incidence falls below the elimination threshold after 40 years, if the reduction is 96% (Figure 2A). Not surprisingly, more infections would be prevented by implementing the universal T&T strategy than by achieving universal access to treatment (Figure 2B): over 20 years it would be 106% more if the reduction in infectivity is 96% or 168% more if the reduction is 85%. Notably, our results show that achieving universal access to treatment could function as a very effective ‘treatment as prevention’ approach if the reduction in infectivity is high, 96%. Under this condition, incidence would be significantly reduced: ∼54% after 20 years and ∼66% after 40 years (Figure 2A). This would prevent ∼4 million infections after 20 years and ∼11 million after 40 years (Figure 2B). However, if the reduction in infectivity is 85% achieving universal access to treatment would have little effect on incidence (dashed black curve, Figure 2A).

Implementing the universal T&T strategy versus achieving universal access to treatment would result in very different treatment patterns (Figure 2C). Under the T&T strategy the number on treatment would peak a few years after implementation at ∼5 million and remain high, even when the epidemic is close to elimination (Figure 2C). After 40 years, ∼2.5 million individuals would be on treatment if the reduction in infectivity is 96% and ∼5.25 million if the reduction is 85%. To achieve universal access the number on treatment would steadily increase over time (Figure 2C). After 40 years it would reach ∼4.5 million if the reduction in infectivity is 96% or ∼6 million if the reduction is 85%. The T&T strategy would initially require treating more individuals each year than would be necessary to achieve universal access to treatment (Figure 2C). This would be for ∼15 years if the reduction in infectivity is 96% or ∼20 years if the reduction is 85%.

**Figure 3:**
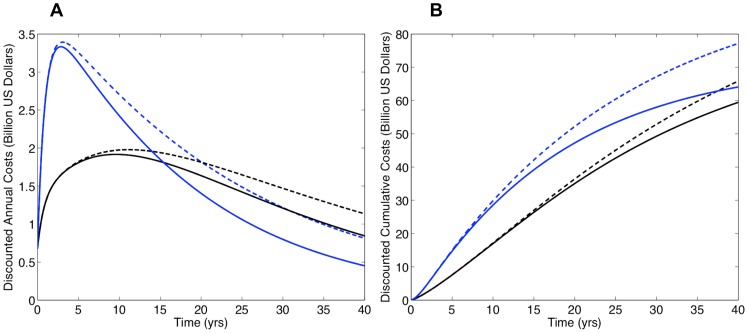
Comparison of the universal ‘test and treat’ (T&T) strategy (blue curves) and achieving universal access to treatment (black curves) in terms of discounted treatment costs. Costs are discounted by 3.5% annually, following Granich *et al.* [10]. The dynamics of acquired and transmitted drug resistance are not included in these simulations. Solid lines denote the case where the treatment-induced reduction in infectivity is 96% and dashed lines denote the case where the reduction is 85%. Panels show (**A**) discounted annual treatment costs over time and (**B**) discounted cumulative treatment costs over time. The dynamics of acquired and transmitted drug resistance are not included in these simulations.

Discounted annual (Figure 3A) and cumulative (Figure 3B) cost curves for South Africa were calculated using the model generated predictions for the numbers on treatment shown in Figure 2C. Since the simulations in Figure 2C did not include the development and transmission of drug-resistant strains of HIV, costs were only calculated based on the need for first-line regimens. Cost curves for the universal T&T strategy are shown in blue and cost curves for universal access to treatment in black. Solid curves are based on the assumption the treatment-induced reduction in infectivity is 96%, dashed curves assume 85%. Since future costs are discounted, annual treatment costs decrease even as the numbers on treatment increase. A cross-over in annual costs occurs at ∼15 years (solid curves) or ∼20 years (dashed curves) (Figure 3A). Notably, the cumulative cost curves never cross over. The cumulative treatment costs for the universal T&T strategy are always greater than the cumulative treatment costs for achieving universal access to treatment. Cumulative costs for both are substantial, even if the reduction in infectivity is 96% (Figure 3B). After 20 years these costs are ∼$48 billion for T&T and ∼$36 billion for universal access to treatment. After 40 years costs are ∼$65 billion for T&T and ∼$60 billion for universal access. If the reduction in infectivity is 85%, cumulative costs are even higher (Figure 3B). After 40 years costs are ∼$78 billion for T&T and ∼$66 billion for universal access. The lower the reduction in treatment-induced infectivity, the higher the treatment costs (Figure 3A and 3B).

**Figure 4:**
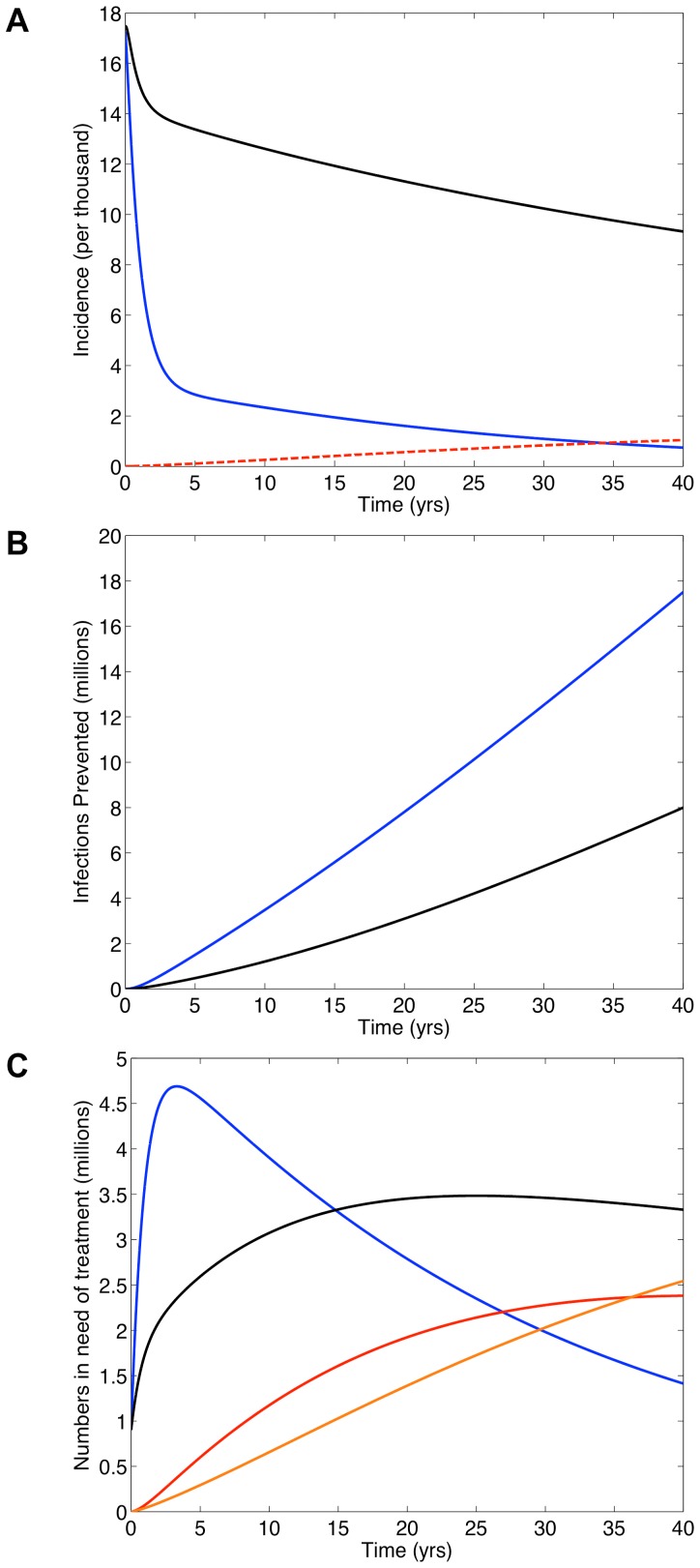
Predictions for South Africa from our transmission model if universal access to treatment is achieved or a universal T&T (with annual testing) strategy is implemented. Dynamics of acquired and transmitted drug resistance are included in these simulations. To generate this figure we assumed a treatment-induced reduction in infectivity of 85%, acquired drug resistance develops in treated individuals at a rate of 3% per year [10] and drug-resistant strains are 50% as transmissible as wild-type strains. Panel (**A**) shows annual incidence over time. The blue curve shows wild-type incidence if the T&T strategy is implemented; black curve shows wild-type incidence if universal access to treatment is achieved. The red dashed curve shows the maximum incidence of transmitted drug resistance if either universal access is achieved or the T&T strategy is implemented. Panel (**B**) shows the number of infections prevented over time for the T&T strategy (blue curve) and if universal access to treatment (black curve) is achieved. Panel (**C**) shows the number of individuals in need of treatment over time. Blue and red curves represent the T&T strategy; the blue curve shows the number of individuals in need of first-line regimens, the solid red curve shows the number in need of second-line regimens. Black and orange curves represent achieving universal access to treatment; the black curve shows the number in need of first-line regimens, the orange curve shows the number in need of second-line regimens.

Figure 4 shows the predictions for South Africa if universal access to treatment is achieved or a universal T&T (with annual testing) strategy is implemented. Simulations were generated from our transmission model with resistance included and based on a treatment-induced reduction in infectivity of 85%. After 40 years, incidence is less than 0.1% under the T&T strategy (blue curve, Figure 4A), which is very similar to incidence predictions when resistance is not included in the model (dashed blue curve, Figure 2A). Therefore, a similar number of infections would be prevented under the T&T strategy whether or not resistance evolves (compare blue curve in Figure 4B with dashed blue curve in Figure 2B). In contrast, the impact that achieving universal access to treatment would have on incidence is very dependent upon whether or not resistance evolves. Without resistance, achieving universal access would have little effect on incidence (dashed black curve, Figure 2A). With resistance, achieving universal access would substantially reduce incidence by ∼45% (black curve, Figure 4A) and prevent ∼8 million infections (black curve in Figure 4B) after 40 years. Notably, under either the T&T strategy or if universal access to treatment is achieved levels of transmitted resistance would remain below 0.1% (dashed red curve, Figure 4A).

**Figure 5:**
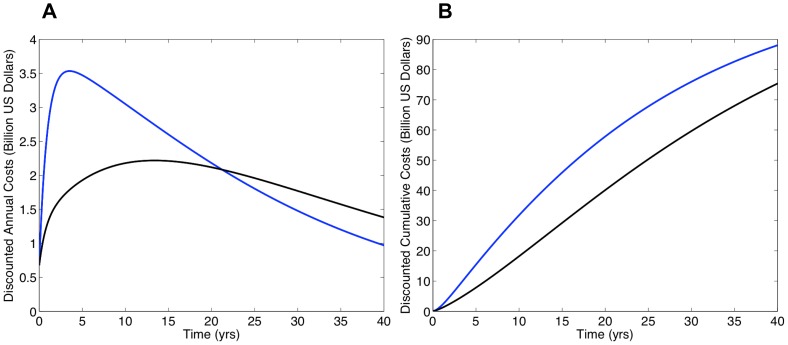
Comparison of the costs of the universal ‘test and treat’ (T&T) strategy (blue curve) and achieving universal access to treatment (black curve) in terms of discounted treatment costs. Costs are discounted by 3.5% annually, following Granich *et al.* [10]. Dynamics of acquired and transmitted drug resistance are included in these simulations. To generate this Figure we assumed a treatment-induced reduction in infectivity of 85%, acquired drug resistance develops in treated individuals at a rate of 3% per year [10] and drug-resistant strains are 50% as transmissible as wild-type strains. Panel (**A**) shows discounted annual treatment costs over time and (**B**) shows discounted cumulative treatment costs over time. Treatment costs include the costs for both first-line and second-line regimens.

If resistance evolves, there would be a great need for second-line regimens. After 20 years, ∼2 million individuals would need second-line regimens if the universal T&T strategy is implemented (red curve, Figure 4C) versus ∼1.5 million if universal access to treatment is achieved (orange curve, Figure 4C). Predicted need for first-line regimens is shown in Figure 4C (blue curve represents T&T, black curve represents universal access to treatment). Discounted annual and cumulative costs for first-line and second-line regimens are given in Figures 5A and 5B, respectively. Peak annual discounted costs under the T&T strategy (blue curve, Figure 5A) reach ∼$3.5 billion within a few years and to achieve universal access to treatment (black curve, Figure 5A) ∼$2 billion after ∼11 years. After 40 years, discounted annual costs are fairly similar: ∼$1 billion (T&T) versus ∼$1.5 billion (universal access). Notably, discounted cumulative treatment costs for the universal T&T strategy (blue curve, Figure 5B) are always greater than the costs to achieve universal access to treatment (black curve, Figure 5B). After 20 years these costs have risen to ∼$59 billion (T&T) versus ∼$40 billion (universal access). After 40 years, discounted cumulative costs rise to ∼$88 billion (T&T) versus ∼$76 billion (universal access).

## Discussion

Our analysis of R_c_ shows if the treatment-induced reduction in infectivity is ≥85%, the HIV epidemic in South Africa could (theoretically) be eliminated by using a universal ‘test and treat’ strategy. These results are in agreement with those of the WHO [5], [10]. Importantly and in contrast to the WHO, our analysis of R_c_ shows if the treatment-induced reduction in infectivity is 96% the HIV epidemic in South Africa could (theoretically) be brought close to elimination by achieving universal access to treatment. Using R_C_ to identify the conditions under which HIV elimination could occur can be informative. However, using R_C_ can also be extremely misleading for four reasons. First, analyzing R_C_ does not provide any indication of how long it would take to achieve elimination; in the case of HIV epidemics it has been shown it could take 50 to 100 years [17]. Second, the analysis does not provide any indication of the number of individuals who would need to be treated to achieve elimination. Third, it does not provide any indication of how long individuals would need to remain on treatment after elimination has been achieved. Fourth, an analysis of R_C_ does not account for the emergence and transmission of drug-resistant strains. When resistance emerges, multiple R_c_'s need to be evaluated in order to determine if elimination is (theoretically) possible [12], [13], [17] In addition, it needs to be understood the conditions that reduce R_C_ to below one (e.g., the degree of viral suppression that reduces infectivity to 96%) would need to be continuously maintained until all of the treated individuals have died. Incidence would increase if the necessary conditions were not continuously maintained. We recommend any analysis of R_C_ that shows HIV elimination is possible should be viewed with great caution.

The numerical simulations generated by our transmission model show a universal T&T strategy with a 96% treatment-induced reduction in infectivity could eliminate HIV in South Africa. This result is in agreement with the WHO [5], [10]. However we predict it would take 40 years, whereas the WHO predicts it would occur within a decade [5], [10]. Also, in contrast to the WHO, our modeling shows attaining universal access to treatment in South Africa would prevent millions of infections and, as well as being a therapeutic strategy, would also function as a very effective ‘treatment as prevention’ strategy. The difference between our results and those of the WHO is because they assume a much shorter survival time on treatment than we do.

Our results have significant implications for evaluating prevention strategies and choosing the optimal combination of these strategies. Several different prevention modalities are now available. As well as ‘treatment as prevention’ clinical studies have also shown pre-exposure prophylaxis, vaginal microbicides and circumcision could be very effective in reducing transmission [16], [18]–[22]. Prevention strategies are generally compared in terms of their effects on transmission and their costs. Our modeling has shown widespread treatment could, in some cases, substantially reduce transmission due to the development of drug-resistant strains of HIV in treated individuals. This is because: (i) some of the treated individuals would be transmitting resistant, rather than wild-type, strains and (ii) resistant strains are less transmissible than wild-type strains. Under these circumstances a ‘treatment as prevention’ strategy would appear very beneficial, but would actually be detrimental as it would have generated a high prevalence of resistance. In addition, our modeling has shown a universal T&T strategy would require millions of individuals to be treated for several decades. Under these conditions cost projections can be misleading. As we have shown, predicted annual treatment costs (since future costs are discounted) could decrease, but the numbers on treatment (and the number of resistant cases) increase. Taken together, our results show it is essential to use multiple evaluation criteria, including sustainability, to compare ‘treatment as prevention’ with other prevention strategies.

Implementing a universal T&T strategy in South Africa would necessitate, almost immediately, treating ∼5 million individuals. This would require substantial financial resources and investment in healthcare infrastructure. Currently, these financial resources are not available. The WHO has argued that implementing a universal T&T strategy in South Africa is worthwhile because it would substantially reduce transmission, and (after 40 years) would cost ∼$10 billion less than achieving universal access to treatment [5], [10]. In contrast, we have estimated implementing a universal T&T strategy in South Africa would cost ∼$12 billion more than achieving universal access to treatment. Our modeling shows the WHO has substantially under estimated the costs of a universal T&T strategy because they have under estimated the need for first-line and second-line treatment regimens. This is the result of (i) assuming an unrealistically short survival time on treatment and (ii) ignoring the risk of resistance. Survival time and resistance will drive costs. Costs for T&T need to be realistically estimated so that fair comparisons can be made with alternative prevention strategies. In addition mathematical models need to be based on realistic, rather than overly optimistic, assumptions so that the true impact of ‘treatment as prevention’ can be determined. Currently only ∼1 million individuals in South Africa receive treatment; ∼1.6 million more are in need of treatment based on the current treatment initiation threshold [23]. Before implementing a universal T&T strategy, which may not be sustainable and hence ethical, we recommend striving to achieve universal access to treatment as quickly as possible. This is desperately needed. Achieving universal access in South Africa would increase the life expectancy of millions of HIV-infected individuals. In addition, it could also function as a very effective ‘treatment as prevention’ approach; preventing 4 million infections in South Africa over the next 20 years.

## Supporting Information

Material S1 **Description of the mathematical model and derivation of the Control Reproduction Number.**
(DOCX)

Figure S1 **Flow diagrams describing the structure of the Granich** ***et al.*** **model** [**1**] **(a) and our mathematical model (Equations 1–10) in the absence of drug resistance (b).** The population is divided into susceptible (**S**), infected and untreated (**I**) and infected individuals receiving first-line therapy (**A**). The relative infectivity in each stage is denoted by λ. Times shown indicate the average period spent in each infected and untreated stage (**I_i_**).
(DOCX)

Figure S2 **Comparison of historical HIV prevalence data for South Africa and historical HIV prevalence generated by our mathematical model.** Our mathematical model (Equations 1–10) is parameterized to account for the pre-treatment era as well as for heterogeneity in sexual behavior. Historical HIV prevalence is based on antenatal clinic data [1], [4], [5].
(DOCX)

Figure S3 **Historical HIV incidence in South Africa generated by our mathematical model.** Our mathematical model (Equations 1–10) is parameterized to account for the pre-treatment era as well as for heterogeneity in sexual behavior [1], [4], [5].
(DOCX)

Table S1 **Parameterization of the mathematical model (Equations 1–10) for implementing the universal ‘test and treat’ (T&T) strategy and achieving universal access to treatment for South Africa.**
(DOCX)
