# Supplementary material for: Universal Access to HIV Treatment versus Universal ‘Test and Treat’: Transmission, Drug Resistance & Treatment Costs
Source: PLoS One. 2012 Sep 5;7(9):e41212. doi: 10.1371/journal.pone.0041212 (PMC3434222; doi:10.1371/journal.pone.0041212)
Supplement: Material S1 — Description of the mathematical model and derivation of the Control Reproduction Number. (DOCX) [file pone.0041212.s001.docx]

**Supplementary Material**

**Universal Access to HIV treatment Versus Universal ‘Test and Treat’: transmission, drug resistance**

**& treatment costs**

Bradley G. Wagner^1^, Sally Blower^1#^

Affiliations: ^1^David Geffen School of Medicine, University of California Los Angeles, Los Angeles, California.

^#^Corresponding author: [sblower@mednet.ucla.edu](mailto:sblower@mednet.ucla.edu)

1. **HIV transmission model structure**

To predict the epidemiological impact of implementing the universal ‘test and teat’ strategy (T&T) and achieving universal access to treatment we used a compartmental, HIV transmission model. The model includes primary, chronic and symptomatic stages of HIV infection, HIV treatment (initiated at different stages of infection), as well as acquired and transmitted drug resistance. The flow diagram showing the structure of the model is given in Figure S1B. This model is a modified version of that of Granich et *al.* [1]. The model is specified by the following set of differential equations:

**
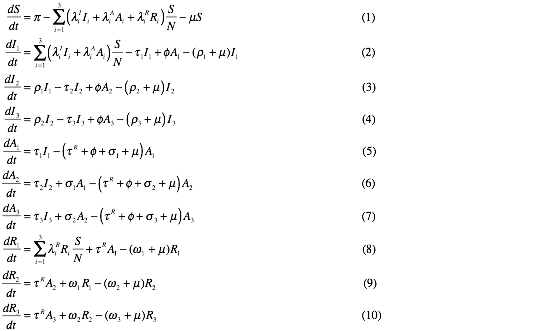
**

Here *S* represents the size of the uninfected population and *N* represents the total population size. The variables *I*_1_, *I*_2_ and *I*_3_ denote the number of HIV-infected individuals in primary, chronic and symptomatic stages of infection. *A*_1_, *A*_2_ and *A*_3_ represent the number of individuals receiving first-line treatment regimens. Individuals initiating treatment during primary infection progress to treatment stage *A*_1_, while those who initiate during chronic infection progress to *A*_2_. Individuals in the symptomatic stage of infection enter the treatment stage *A*_3_. The variables *R*_1_, *R*_2_ and *R*_3_ denote the number of individuals infected with drug-resistant strains. For the T&T strategy, the sum *R_1_+ R_2_ +R_3_* gives the number of individuals receiving second-line therapy (see Table S1 for parameterization). For achieving universal access to treatment, *R_3_* represents the number of individuals receiving second-line treatment regimens while *R_1_* and *R_2_* represent the number of individuals with transmitted resistance who have not yet begun treatment (see Table S1 for parameterization).

Individuals enter the sexually active population at a rate *π* and die at a per capita rate *μ*. Treatment rates in primary, chronic, and symptomatic stages of infections are represented by *τ*_1_, *τ*_2_ and *τ*_3_. Untreated individuals progress from primary to chronic infection at a rate *ρ*_1_ and from chronic infection to symptomatic infection at a rate *p*_2_. Symptomatic individuals die from AIDS at a rate *ρ*_3_. Individuals receiving treatment discontinue or interrupt treatment at a rate *φ*. Treated individuals who initiate treatment during primary infection progress to the second treatment stage at a rate *σ*_1,_ and those in the second stage to the third at a rate σ_2_. Once in the third and final treatment stage individuals die at a rate *σ*_3_. Treated individuals acquire drug resistance at an average rate τ^R^. Drug-resistant individuals progress through the stages *R*_1_, *R*_2_ and *R*_3_ at rates *ω*_1_, *ω*_2_ and *ω*_3_ respectively. The infectivity per unit time of individuals in stages *I*_i_, *A*_i_ and *R*_i_ is given, respectively, by *λ* _i_^I^, *λ* _i_^A^ and *λ*_i_^R^. For specific parameterizations for achieving universal access to treatment and implementing the T&T strategy for South Africa see Table S1.

**2. Computation of the Control Reproduction Number (R_c_)**

We computed the Control Reproduction Number R_c_ for the transmission model in the absence of drug resistance (i.e., for the model specified by equations 1 through 7 setting *τ* ^R^, *λ*_i_^R^=0 for *i*=1,2,3). R_c_ may be computed (analytically) as the spectral radius (i.e., maximum modulus of the eigenvalues) of the next generation matrix *FV*^-1^ [2,3], where *F* and *V* are given by the following expressions:


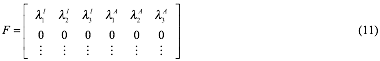


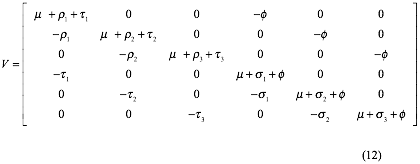


Note that *FV^-1^* has only one nonzero eigenvalue with corresponding eigenvector .


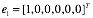


The Control Reproduction Number may be straightforwardly expressed in a biologically interpretable manner as


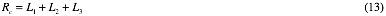


where can be interpreted as the Control Reproduction Number for a model including only the (infected) stages and weighted by the probability that an individual has progressed to these stages before dying. The are given as follows:

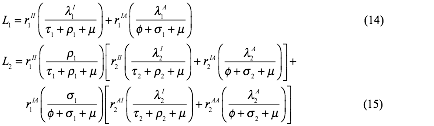


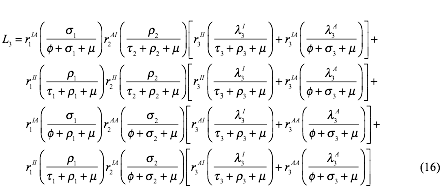


where,


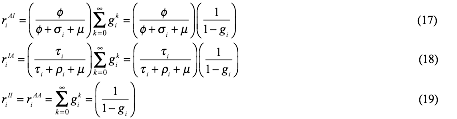


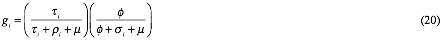


The terms give the probability that an individual in class begins treatment and subsequently interrupts it (or vice versa). Similarly, the represent the sum of the probabilities for all possible paths an individual may take from to by successively interrupting and then resuming treatment (and vice versa ).


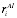

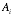

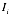

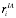


**References:**

1. Granich RM, Gilks CF, Dye C, De Cock KM, Williams BG (2009) Universal voluntary HIV testing with immediate antiretroviral therapy as a strategy for elimination of HIV transmission: a mathematical model. Lancet 373: 48-57.

2. Diekmann O, Heesterbeek JAP, Metz JAJ (1990) On the definition and the computation of the basic reproduction ratio R0 in models for infectious diseases in heterogeneous populations. J Math Biiol 28: 365-382.

3. van den Driessche P, Watmough J (2002) Reproduction numbers and sub-threshold endemic equilibria for compartmental models of disease transmission. Math Biosci 180: 29-48.

4. National Department of Health (2007) The National Antenatal Sentinel HIV and Syphilis Prevalence Survey, South Africa, 2006. <http://www.doh.gov.za/list.php?type=HIV%20and%20AIDS&year=2007>. Accessed 2012 Aug 13.

5. National Department of Health (2011) The National Antenatal Sentinel HIV and Syphilis Prevalence Survey, South Africa, 2010. <http://www.doh.gov.za/docs/reports/2011/hiv_aids_survey.pdf>. Accessed 2012 Aug 13.
