# Supplementary figures and images for: Universal Access to HIV Treatment versus Universal ‘Test and Treat’: Transmission, Drug Resistance & Treatment Costs
Source: PLoS One. 2012 Sep 5;7(9):e41212. doi: 10.1371/journal.pone.0041212 (PMC3434222; doi:10.1371/journal.pone.0041212)

**Figure S1**

**
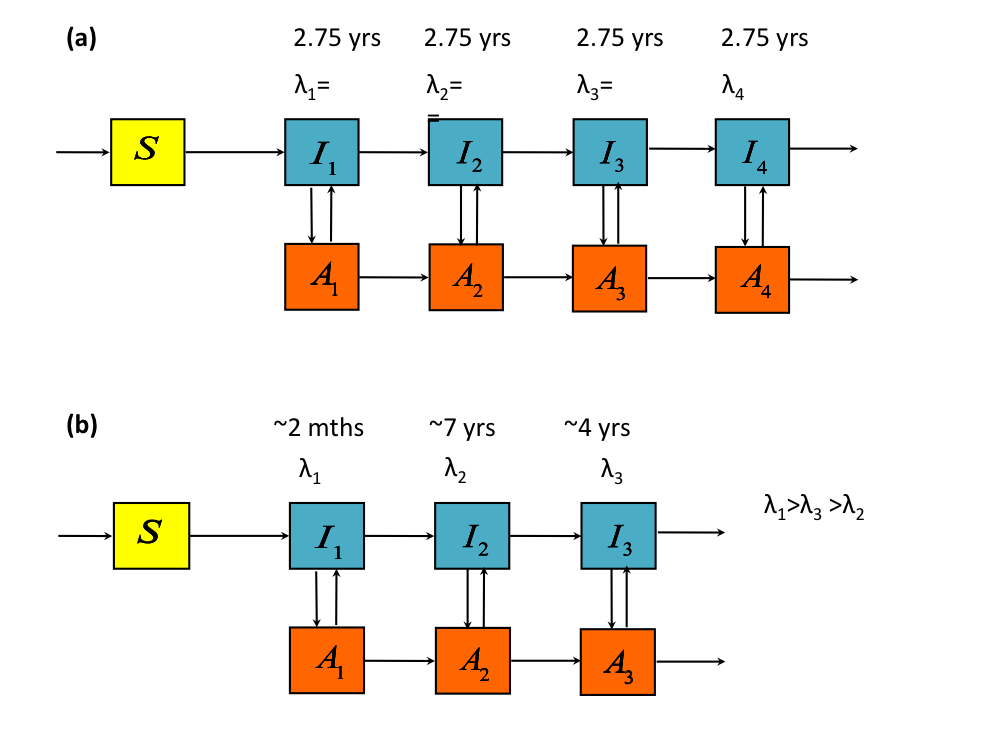
**

Supplement: Figure S1 — Flow diagrams describing the structure of the Granich et al. model [1] (a) and our mathematical model (Equations 1–10) in the absence of drug resistance (b). The population is divided into susceptible (S), infected and untreated (I) and infected individuals receiving first-line therapy (A). The relative infectivity in each stage is denoted by λ. Times shown indicate the average period spent in each infected and untreated stage (Ii). (DOCX) [file pone.0041212.s002.docx]

**Figure S2**


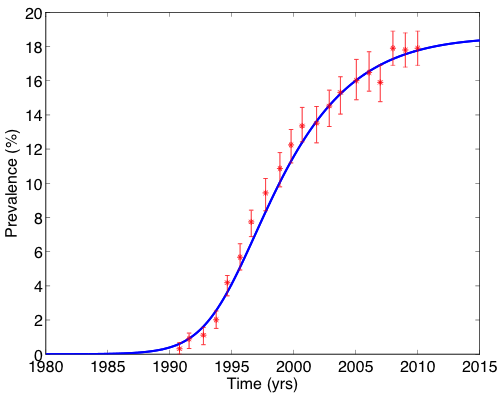

Supplement: Figure S2 — Comparison of historical HIV prevalence data for South Africa and historical HIV prevalence generated by our mathematical model. Our mathematical model (Equations 1–10) is parameterized to account for the pre-treatment era as well as for heterogeneity in sexual behavior. Historical HIV prevalence is based on antenatal clinic data [1], [4], [5]. (DOCX) [file pone.0041212.s003.docx]

**Figure S3**


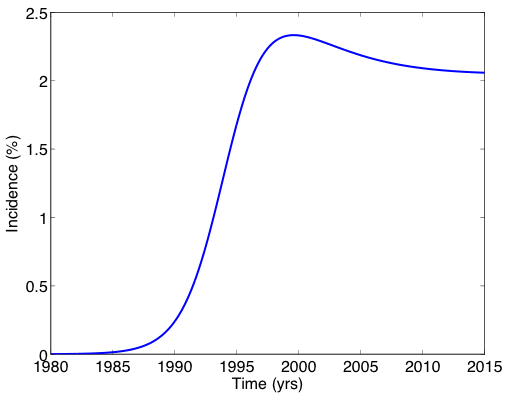

Supplement: Figure S3 — Historical HIV incidence in South Africa generated by our mathematical model. Our mathematical model (Equations 1–10) is parameterized to account for the pre-treatment era as well as for heterogeneity in sexual behavior [1], [4], [5]. (DOCX) [file pone.0041212.s004.docx]
