# Supplementary material for: Universal Access to HIV Treatment versus Universal ‘Test and Treat’: Transmission, Drug Resistance & Treatment Costs
Source: PLoS One. 2012 Sep 5;7(9):e41212. doi: 10.1371/journal.pone.0041212 (PMC3434222; doi:10.1371/journal.pone.0041212)
Supplement: Table S1 — Parameterization of the mathematical model (Equations 1–10) for implementing the universal ‘test and treat’ (T&T) strategy and achieving universal access to treatment for South Africa. (DOCX) [file pone.0041212.s005.docx]

**Table S1:**

| **Parameter** | **Description** | **T&T** | | **Universal Access** |
| --- | --- | --- | --- | --- |
| π | Rate of joining the sexually active population | 582,000 yr^--1^ | | |
| 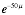 | Probability of a sexually active, HIV-negative individual surviving 50 years or more | 70% | | |
| 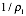 | Mean duration of primary infection | 2 months | | |
| 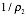 | Mean duration of chronic infection | 7.3 years | | |
| 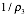 | Mean duration of symptomatic infection | 3.5 years | | |
| 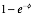 | Probability of interrupting treatment per year | 2% | | |
| 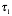 | Per capita treatment rate during primary infection | 1.0 yr^-1^ | | 0 yr^-1^ |
| 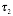 | Per capita treatment rate during chronic infection  (>350 cells/μL) | 1.0 yr^-1^ | | 0 yr^-1^ |
| 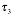 | Per capita treatment rate during symptomatic infection (<350 cells/μL) | 1.0 yr^-1^ | | |
| 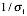 | Mean duration of treatment in stage A_1_ | 2 months | | |
| 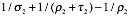 | Mean life-years gained through early treatment versus treatment at 350 cells/μL | 6 years | | |
| 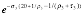 | Probability of gaining more than 20 years of additional life, if treatment is initiated at 350 cells/μL | 62% | | |
| 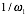 | Mean duration of primary infection if infected with resistant strain | 2 months | | |
| 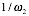 | Mean duration of chronic infection if infected with resistant strain | 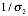 | | 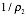 |
| 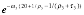 | Probability of gaining more than 20 years of additional life, if treatment is initiated at 350 cells/μL and individual is infected with resistant strain | 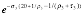 | | |
| 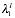 | Transmission rate: primary infection | 0.51 yr^-1^ | | |
| 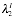 | Transmission rate: chronic infection | 0.11 yr^-1^ | | |
| 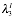 | Transmission rate: symptomatic infection | 0.15 yr^-1^ | | |
| 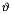 | Reduction in infectivity due to treatment | 85%-96% | | |
| 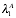 | Transmission rate: treatment stage A_1_ | 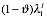 | | |
| 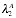 | Transmission rate: treatment stage A_2_ | 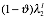 | | |
| 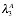 | Transmission rate: treatment stage A_3_ | 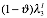 | | |
| 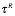 | Rate of acquiring resistance in the treated population | 3% per year | | |
| 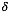 | Reduction in transmissibility of resistant strains | 50% | | |
| 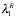 | Transmission rate for resistant strains: stage R_1_ | 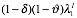 | 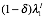 | |
| 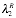 | Transmission rate for resistant strains: stage R_2_ | 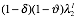 | 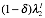 | |
| 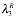 | Transmission rate for resistant strains: stage R_3_ | 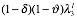 | | |
